# Molecular identification and genetic diversity of open reading frame 7 field isolated porcine reproductive and respiratory syndrome in North Sumatera, Indonesia, in the period of 2008-2014

**DOI:** 10.14202/vetworld.2015.875-880

**Published:** 2015-07-16

**Authors:** Faisal Faisal, Rini Widayanti, Aris Haryanto, Charles Rangga Tabu

**Affiliations:** 1 Department of Veterinary Science, Faculty of Veterinary Medicine, Gadjah Mada University, Yogyakarta, Indonesia; 2 Department of Molecular Biology, Animal Disease Investigation Centre of Medan, North Sumatera, Indonesia; 3 Department of Biochemistry, Faculty of Veterinary Medicine, Gadjah Mada University, Yogyakarta, Indonesia; 4 Department of Pathology, Faculty of Veterinary Medicine, Gadjah Mada University, Yogyakarta, Indonesia

**Keywords:** Indonesia, North Sumatera, open reading frame 7, porcine reproductive and respiratory syndrome virus

## Abstract

**Aim:**

Molecular identification and genetic diversity of open reading frame 7 (ORF7) of field isolated porcine reproductive and respiratory syndrome virus (PRRSV) in North Sumatera, Indonesia, in the period of 2008-2014.

**Materials and Methods:**

A total of 47 PRRSV samples were collected from the death case of pigs. The samples were collected from different districts in the period of 2008-2014 from North Sumatera province. Two pairs of primer were designed to amplify ORF7 of Type 1 and 2 PRRSV based on the sequence of reference viruses VR2332 and Lelystad. Viral RNAs were extracted from samples using PureLink™ micro-to-Midi total RNA purification system (Invitrogen). To amplify the ORF7 of PRRSV, the synthesis cDNA and DNA amplification were performed by reverse transcription polymerase chain reaction (RT-PCR) and nested PCR method. Then the DNA sequencing of PCR products and phylogenetic analysis were accomplished by molecular evolutionary genetics analysis version 6.0 software program.

**Results:**

RT-: PCR and nested PCR used in this study had successfully detected of 18 samples positive PRRS virus with the amplification products at 703bp and 508bp, respectively. Sequencing of the ORF7 shows that 18 PRRS viruses isolated from North Sumatera belonged to North American (NA). JXA1 Like and classic NA type viruses. Several mutations were detected, particularly in the area of nuclear localization signal (NLS1) and in NLS2. In the local viruses, which were related closed to JXA1 virus; there are two differences in amino acids in position 12 and 43 of ORF7. Our tested viruses showed that the amino acid positions 12 and 43 are Asparagine and Arginine, while the reference virus (VR2332, Lelystad, and JXA1) occupied both by Lysine. Based on differences in two amino acids at position 12 and 43 showed that viruses from North Sumatera has its own uniqueness and related closed to highly pathogenic PRRS (HP-PRRS) virus (JXA1).

**Conclusion:**

The results demonstrated that North Sumatera type PRRS virus has caused PRRS outbreaks in pig in North Sumatera between 2008 and 2014. The JAX1 like viruses had unique amino acid residue in position 12 and 43 of asparagine and lysine, and these were genetic determinants of North Sumatera viruses compared to other PRRS viruses.

## Introduction

Porcine reproductive and respiratory syndrome virus (PRRSV) is an infectious disease in pigs which is caused by a virus and brings about an economic loss in pig breeding industry [1]. The first isolation of Type 1 PRRS virus (European) is called Lelystad virus, which was found in the Netherlands, and Type 2 PRRS virus (North American [NA]), called VR-2323 virus, was isolated in the United States in 1992 [2,3]. The two types of virus belong to RNA virus, enveloped, belong to Arteriviridae family, short chain, and no segment [2].

In 2006, PRRS virus became the important disease in China because of its high morbidity and mortality, which caused the death of millions of pigs. The result of the analysis on clinical symptoms, pathogenicity, and molecular virus showed that this virus was very different from classic PRRS virus so that this virus was then known as highly pathogenic PRRS (HP-PRRS) [4,5]. HP-PRRS has recently been reported in Thailand, Vietnam, Laos, Cambodia, Myanmar, Philippines, and Russia [6-8].

Open reading frame 7 (ORF7) had 123-128 amino acid which consists of N-terminal RNA binding domain and C-terminal dimerization domain [9]. The ORF7 encodes nucleocapsid (N) protein, is frequently performed because it is highly immunogenic in pigs and has an important role in virus virulence and diagnosis [10]. N protein is expressed abundantly and has high immunogenicity so that this protein is generally used as material in diagnostic test [11]. Glycoprotein is a conserved area so that it is frequently used for diagnostic application such as reverse transcription polymerase chain reaction (RT-PCR) and RT-qPCR [5,12,13]. The N protein has also been used for investigating genetic variation and phylogenetic relationships among PRRSV isolates [14,15].

In Indonesia, PRRS disease has not been known by many people. PRRS virus infection occurred in North Sumatera in 2008, but official information about this disease was difficult to obtain. The limitation of the data of the dynamic of PRRS disease in Indonesia had encouraged the researcher to conduct a study on it. In the present study, we determined the complete ORF7 sequences of 18 PRRS viruses from North Sumatera Province. The aims of this study were (1) To find out the genetic database for the ORF7 gene in North Sumatera PRRSV viruses; (2) to compare North Sumatera PRRSV ORF7 sequences with the ORF7 sequences of the reference viruses, (3) to determine the phylogenetic relationships between North Sumatera PRRSVs and reference viruses.

## Materials and Methods

### Ethical approval

This research was conducted after approval by institutional research committe of Gadjah Mada University.

### Sample collection

A total of 47 samples were collected from the death case of pigs. The samples were collected from different districts in the period of 2008-2014 in North Sumatera.

### Primers

In this study, two pairs of primer which was able to amplify ORF7 in Type 1 and 2 PRRS viruses. The two pairs were designed by using isolate reference VR2332, Lelystad, and also used multi alignment from the sequences of the two virus types, which were available in GenBank data. Primer3 software (http://primer3.ut.ee/) was used to help in designing the primers for this study. The specific primers ORFF-5’-GCCCTAATTGACTAGGTGACT-‘3 and ORFR-5’-GCCCTAATGACTAGGTGACT-‘3, were designed on the ORF6 and 3’UTR sequences to generate a 703 bp fragment including the whole ORF7 sequence. The second PCR was performed using the primers described by Guarino [16].

### Synthesis of cDNA and external PCR

Total RNA was isolated from samples by PureLink™ micro-to-Midi total RNA purification system (Invitrogen) according to the instructions of the manufacturer. In RT, we used of SuperScript III First-Strand Synthesis (Invitrogen). RT contained the following ingredients: 5 µl of RNA, 1-µl (50 µM) primer oligo (dT) 20, 1-µl (10 mM) dNTP, and 4 µl diethylpyrocarbonate. The RNA was denatured at 65°C for 5 min and then cooled on ice. Master mix consisting of 10 µl cDNA synthesis mix was added at 10 µl sample and then incubated in a thermal cycler at 50°C for 50 min, 85°C for 5 min, and then stored at −20°C.

The PCRs contained the following ingredients, 2 µl sample, 2 µl (20 µM) of forward primer, 2 µl (20 µM) of the reverse primer, and 45 µl Platinum^®^ PCR SuperMix. The PCRs were run as follows, pre-denaturation at 95°C for 2 min, and 40 cycles of denaturation at 95°C for 45 s, annealing at 50°C for 1-min, extension at 72°C for 45 s, and final extension at 72°C for 5 min.

### Nested PCR

For nested PCR, we used KAPA2G™ Fast Ready Mix 2X (KapaBiosystems). The PCRs contained the following ingredients, 12.5 µl of 2x KAPA2G Fast Ready Mix, 1.25 µl (10 µM) of forward primer, 1.25 µl (10 µM) of reverse primer, 5 µl of PCR product, and the addition of RNA-se free water until 25 µl. The PCRs were run as follows, pre-denaturation at 95°C for 2 min, and 40 cycles of denaturation at 95°C for 20 s, annealing at 49°C for 20 s, extension at 72°C for 30 s, and final extension at 72°C for 3 min.

### Sequencing of nucleotide

PCR products were sequenced using the Big Dyes TN kit (Applied Biosystems, USA) following the manufactories instructions, run, and analyses on an ABI PRISM^®^ 3700 DNA Analyzer. All products were sequenced in both directions. Identification of the resulting nucleotide sequence of ORF7 in local viruses was tested with BLAST in order to assure the specificity of the PCR. Multiple nucleotide ORF7 virus sequence was aligned by using Clustal W and compared with some isolate references (V2332, Lelystad, Ch-Ia, MLV, and JXA1).

### Phylogenetic analysis

To analyze the ORF7 genes of isolates from North Sumatera, Indonesia, Phylogenetic reconstructions were generated using the neighbor-joining method by the computer program Molecular Evolutionary Genetics Analysis Version 6 based on the formulas of Kimura 2-parameter. The robustness of the phylogenetic analysis was determined by bootstrap analysis with 1000 replications.

## Results and Discussion

### PCR

**Figure-1:**
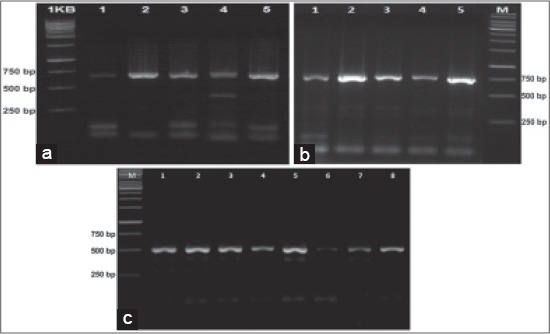
The result of amplification of two-step polymerase chain reaction (PCR) and nested PCR open reading frame 7 (ORF7) in agarose. (a) and (b), ORF7 was amplified in band 703bp, using ORFF and ORFR primers, (c) ORF7 was amplified with nested PCR in amplicons 508bp, using P71F and P7IR primers.

About 10 of 47 samples were detected PRRSV-positive using external primers with the amplicon size of 703bp. Of the 37 samples, which found negative by the external primer, 8 were PRRSV-positive by nested PCR using P71F and P71R primers with the amplicon size of 508bp. The result of amplicons ORF7 from Two-Step PCR and nested PCR through gel electrophoresis could be seen in Figure-1.

### Sequence analysis

In this study, there were 18 complete sequences of ORF7, which came from 9 districts in North Sumatera. The specific primers ORFF and ORFR were designed to amplify gene target, which flanks from the partial of ORF6 to 3’UTR as long as 703 bp of DNA fragment, this flanking region including the whole of ORF7 sequence. The whole ORF7 of the North Sumatera PRRSV sequences have the same nucleotides length in size of 369nt, which encoded 123 amino acids. There was no deletion and insertion in all local virus sequences. The nucleotide analysis indicated several points of silent mutations or missense mutations, compared with classic PRRS viruses (VR2332, MLV, and CH-1a) and HP-PRRS virus (JXA1).

**Figure-2:**
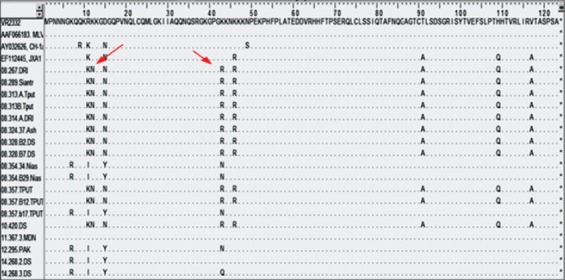
Multi alignment of 18 sequences of an amino acid of local viruses. Local viruses, which were related close to highly pathogenic porcine reproductive and respiratory syndrome (JXA1) reference virus, were found a unique amino acid. The two amino acids were placed by aspartic acid (12N) and arginine (43R) (red arrow).

N protein of PRRS virus has been recognized to have some areas [17]. The areas of nuclear localization signal 1 (NLS1) and NLS2 were the areas where amino acid mutations frequently occurred [17]. In North Sumatera PRRS viruses, amino acid mutations found in position 7 (K7R), 11 (R11K or R11I), 12 (K12N), and 15 (D15N or D15Y) areas by which these positions have been previously reported as high variable region [17]. NLS2 area also underwent mutation points; the mutation point occurred in the position of 43 (K43R or K43N), 46 (K46R). The domain area IV, which was formed by amino acids 69-112, was one of the important antigenic domain areas in ORF7 [17], the mutation point occurred in the position of 91 (T91A) and 109 (H109Q). In the domain area V, in the position of amino acid 117, 11 local viruses were placed by Alanine, and 7 local viruses were placed by Valine. This area included the high variable area of N protein. Some mutation found in several North Sumatera viruses that indicate close related with JXA1 (K46R, T91A, H109Q, and V117A) but in the North Sumatera viruses, which were related closed to JXA1 virus, there are two differences amino acids in position 12 and 43. The two amino acids were placed by aspartic acid and arginine (Figure-2).

N protein in PRRS virus had multifunction [18]; one of them was functioned as phosphoprotein Serine [19]. Phosphorylation in N protein occurred in the amino acid position 120 which was by Serine and all local viruses were placed by serine in this position (Table-1). Type 2 virus had three Cysteine residues, which were conserved; the three residues were located in the position of C23, C75, and C90, respectively [20]. These three positions were very important for virus effectiveness, and all local viruses indicated cysteine in this position (Table-1).

**Tabel 1:** Amino acid variation in the important sites of ORF7.

Virus name	Amino acid variation of ORF7											

	A			B	C			D				E
				
	7	12	43	30-37	23	75	90	46	91	109	117	120
VR2332	K	K	K	IAQNQSR	C	C	C	K	T	H	V	S
MLV	-	-	-	-	-	-	-	-	-	-	-	-
Ch-1a	-	-	-	-	-	-	-	-	-	-	-	-
JXA1	-	-	-	-	-	-	-	R	A	Q	A	-
08.267 Dairi	-	N	R	-	-	-	-	R	A	Q	A	-
08.289 Siantar	-	N	R	-	-	-	-	R	A	Q	A	-
08.313A Taput	-	N	R	-	-	-	-	R	A	Q	A	-
08.313B Taput	-	N	R	-	-	-	-	R	A	Q	A	-
08.314A Dairi	-	N	R	-	-	-	-	R	A	Q	A	-
08.324.37 Ash	-	N	R	-	-	-	-	R	A	Q	A	-
08.328.B2 DS	-	N	R	-	-	-	-	R	A	Q	A	-
08.328.B7 DS	-	N	R	-	-	-	-	R	A	Q	A	-
08.354.34 Nias	R	-	N	-	-	-	-	-	-	-	-	-
08.354.B29 Nias	R	-	N	-	-	-	-	-	-	-	-	-
08.357 Taput	-	N	R	-	-	-	-	R	A	Q	A	-
08.357.B12 Taput	-	N	R	-	-	-	-	R	A	Q	A	-
08.357.B17 Taput	R	-	N	-	-	-	-	-	-	-	-	-
10.420 DS	-	N	R	-	-	-	-	R	A	Q	A	-
11.367.3 MDN	-	-	-	-	-	-	-	-	-	-	-	-
12.295 PAK	R	-	N	-	-	-	-	-	-	-	-	-
14.268.2 DS	R	-	-	-	-	-	-	-	-	-	-	-
14.268.3 DS	R	-	Q	-	-	-	-	-	-	-	-	-

Abbreviation amino acids base on one letter symbols, A=Unique Asam Amino (HP-PRRS like), B=Binding domain with fibrillarin, C=Cysteine conserved, D=Amino acids similar to JXA1, E=Phosphorylation site, ORF7=Open reading frame 7, HP-PRRS=Highly pathogenic porcine reproductive and respiratory syndrome

N protein in Type 2 virus had amino acid as binding domain place with fibrillarin in the position of amino acid 30-37 (IAQQNQSR). The result of the sequence of all local viruses, which indicated domain binding position with fibrillarin had conserved site (Table-1). The interesting thing of local viruses, which had the closeness to JXA1 virus, was that local viruses had unique amino acid; namely, in the position amino acid 12 and 43, were placed by Asparagine and Arginine. The virus which came from China (JXA1 and Ch-1a) and classic virus (VR 2332 and MLV) both positions were filled by Lysine. These two amino acids could be used as the marker of HP-PRRS virus which came from North Sumatera. Local viruses, which had the closeness to the classic virus in this position, were filled by Lysine (12K) and Aspartic Acid (43N). Pathogenicity of PRRS virus which also had its own characteristics had been reported in several countries [21-24].

### Phylogenetic analysis

**Figure-3:**
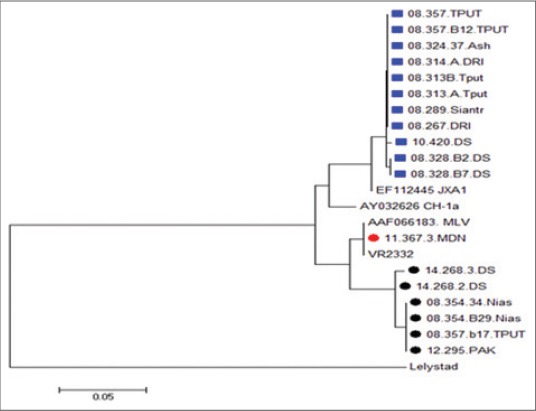
Phylogenetic tree, based on nucleotide sequences of open reading frame 7 local virus and some isolate references. Local viruses were distributed to 2 groups. Blue: Highly pathogenic porcine reproductive and respiratory syndrome cluster, black and red: classic virus cluster. Bootstrap values were calculated using 1000 replicates of the alignment.

Phylogenetic analysis demonstrated that the 18 North Sumatera PRRS viruses were divided into two groups (Figure-3). The first was the viruses that genetically close related to JXAI virus with the viruses shared 91.6-99.1% in the level of nucleotide and 93.4-98.3% level amino acids. High phylogenetic relatedness was found amongst the viruses belonging to the first group in this study (11 viruses in 2008). Both nucleotide and amino acid sequence identities of these viruses were high (97.5-99.1%), indicative of genetic homogeneity. The source of virus transmission most probably came from HP-PRRS China virus in 2006 which spread throughout Asia, including Southeast Asia, and one of them was Indonesia. This was supported by the close relatedness of North Sumatera PRRS viruses to HP-PRRS (JXA1) virus.

The second group was considered of viruses that clustered in the same lineage with the VR2332 virus. The virus shared 93.0-100% nucleotide similarities in the ORF7 and 93.4-100% in the deduced amino acid sequences compared with VR 2332 virus. This indicates that Type 2 PRRS virus had spread widely in North Sumatera Province. The sequences of ORF7 compared with modified live vaccine (MLV) virus, showed 93.0-100% similarity in both nucleotide and amino acid sequences. High phylogenetic relatedness was found among the one virus (11.363.3 Medan) belonging to the second group in this study. The similarity with comparison with MLV virus was about 100% in both nucleotide and amino acid. This virus was a possibility that the vaccine virus has been circulating in North Sumatera.

The existence of North Sumatera viruses was related close to classic virus VR2332 and MLV, which indicated that there was the possibility for the virus which came from vaccine had caused infection in breeding pigs in North Sumatera. The implementation of vaccination, which was not in line with the rule or vaccinating sick animals, would bring about sickness in breeding pigs, which would eventually the incidence of PRRS case occurrence in pigpens and spread to other pigpens. Geographically, Nias District was bordered with the Indian Ocean and the mainland of Sumatera, but virus, which came from Nias and several other local viruses were close to the vaccine virus. Another assumption about the existence of the classic virus was that PRRS virus had long settled in North Sumatera, along with the case of HP-PRRS China virus which spread to Southeast Asia, including North Sumatera. At the same time, the classic virus was also able to infect pigs, which brought about sickness. In order to know more about this case, it was necessary to study epidemiology deeply so that this case could be solved properly.

## Conclusion

After analyzing local viruses which were found in North Sumatera, there were some important things, which could be concluded. First, PRRS virus, which spread in North Sumatera was included in Type 2 PRRS virus (NA); second, in its development, this virus was divided into two groups: Virus group which was close to HP-PRRS virus and virus group which was close to PRRS classic virus; and third, in the HP-PRRS virus group there were amino acids 12N and 43R which could be used as the marker of PRRS virus which came from North Sumatera. The two amino acids were not found in other virus references and HP-PRRS China viruses. Further profound studies in various aspects could be conducted on these local viruses, particularly the function of amino acids 12N and 43R as one of virus virulence factors.

## Author’s Contributions

FF was responsible for the collection of samples ADIC Medan at 2008-2014. AH did the PCR identification and RW phylogenic analysis. CRT drafted and revised the manuscript. All authors read and approved the final manuscript.
